# Integrative transcriptome and metabolome analysis reveals the mechanisms of light-induced pigmentation in purple waxy maize

**DOI:** 10.3389/fpls.2023.1203284

**Published:** 2023-08-15

**Authors:** Yuan Lu, Yao Yu, Yanfang Xuan, Ayiguli Kari, Caixia Yang, Chenyu Wang, Chao Zhang, Wei Gu, Hui Wang, Yingxiong Hu, Pingdong Sun, Yuan Guan, Wenshuai Si, Bing Bai, Xuecai Zhang, Yunbi Xu, Boddupalli M. Prasanna, Biao Shi, Hongjian Zheng

**Affiliations:** ^1^Crop Breeding and Cultivation Research Institute, Shanghai Academy of Agricultural Sciences, Shanghai, China; ^2^CIMMYT-China Specialty Maize Research Center, Shanghai Academy of Agricultural Sciences, Shanghai, China; ^3^Shanghai Engineering Research Center of Specialty Maize, Shanghai Academy of Agricultural Sciences, Shanghai, China; ^4^Key Laboratory of Germplasm Innovation and Genetic Improvement of Grain and Oil Crops (Co-construction by Ministry and Province), Ministry of Agriculture and Rural Affairs, Shanghai, China; ^5^Institute for Agri-Food Standards and Testing Technology, Shanghai Academy of Agricultural Sciences, Shanghai, China; ^6^International Maize and Wheat Improvement Center (CIMMYT), Texcoco, Mexico; ^7^Institute of Crop Sciences, Chinese Academy of Agricultural Sciences, Beijing, China; ^8^International Maize and Wheat Improvement Center (CIMMYT), Nairobi, Kenya; ^9^Shanghai Key Laboratory of Agricultural Genetics and Breeding, Shanghai, China

**Keywords:** waxy maize, anthocyanin, light, transcriptomics, metabolomics

## Abstract

**Introduction:**

Waxy maize, mainly consumed at the immature stage, is a staple and vegetable food in Asia. The pigmentation in the kernel of purple waxy maize enhances its nutritional and market values. Light, a critical environmental factor, affects anthocyanin biosynthesis and results in pigmentation in different parts of plants, including in the kernel. SWL502 is a light-sensitive waxy maize inbred line with purple kernel color, but the regulatory mechanism of pigmentation in the kernel resulting in purple color is still unknown.

**Methods:**

In this study, cyanidin, peonidin, and pelargonidin were identified as the main anthocyanin components in SWL502, evaluated by the ultra-performance liquid chromatography (UPLC) method. Investigation of pigment accumulation in the kernel of SWL502 was performed at 12, 17, and 22 days after pollination (DAP) under both dark and light treatment conditions via transcriptome and metabolome analyses.

**Results:**

Dark treatment affected genes and metabolites associated with metabolic pathways of amino acid, carbohydrate, lipid, and galactose, biosynthesis of phenylpropanoid and terpenoid backbone, and ABC transporters. The expression of anthocyanin biosynthesis genes, such as *4CL2*, *CHS*, *F3H*, and *UGT*, was reduced under dark treatment. Dynamic changes were identified in genes and metabolites by time-series analysis. The genes and metabolites involved in photosynthesis and purine metabolism were altered in light treatment, and the expression of genes and metabolites associated with carotenoid biosynthesis, sphingolipid metabolism, MAPK signaling pathway, and plant hormone signal transduction pathway were induced by dark treatment. Light treatment increased the expression level of major transcription factors such as *LRL1*, *myc7*, *bHLH125*, *PIF1*, *BH093*, *PIL5*, *MYBS1*, and *BH074* in purple waxy maize kernels, while dark treatment greatly promoted the expression level of transcription factors *RVE6*, *MYB4*, *MY1R1*, and *MYB145*.

**Discussion:**

This study is the first report to investigate the effects of light on waxy maize kernel pigmentation and the underlying mechanism at both transcriptome and metabolome levels, and the results from this study are valuable for future research to better understand the effects of light on the regulation of plant growth.

## Introduction

Waxy maize (*Zea mays* L.) is one of the main fresh-eating maize types with many excellent characteristics in terms of starch composition and economic value. Purple waxy maize possesses high nutritional and economic value because it is rich in anthocyanins (Kim et al., 2020). The market has different needs from different colors of maize (De Groote and Kimenju, 2012). Purple waxy maize is famous for its high anthocyanin content, which is much higher than that in other varieties of maize (Paulsmeyer et al., 2017). The pericarp of purple maize is heavily pigmented with anthocyanins, which act as powerful antioxidants (Aoki et al., 2002; Yang et al., 2009; Yang and Zhai, 2010). Several studies support the role of anthocyanins in suppressing free radicals and thereby helping prevent and/or treat certain diseases such as atherosclerosis, diabetes, hypertension, inflammation, cancer, and also aging (Liu et al., 2012). In addition, anthocyanins are a major class of flavonoids that are vital for plant growth and coloration (Ilieva et al., 2016; Moro et al., 2017). Therefore, the pigmentation of purple waxy maize is an important factor to determine maize quality and economic value.

In plants, the anthocyanin biosynthesis pathway is regulated by several structural genes and regulatory transcriptional factors which play a significant role in the production of diverse anthocyanin components (Ming et al., 2021). In maize, anthocyanins are initially biosynthesized from phenylpropanoid metabolism (Petroni and Tonelli, 2011; Sharma et al., 2012). Phenylalanine ammonia-lyase (*ZmPAL*), cinnamic acid 4-hydroxylase (*ZmC4H*), and 4-coumarate CoA ligase (*Zm4CL*) are genes involved in the phenylpropanoid pathway, inducing the first enzymatic step in the flavonoid pathway (Yuan et al., 2019). The flavonoid biosynthetic genes encode enzymes of chalcone synthase (CHS), chalcone isomerase (CHI), and flavanone 3-hydroxylase (F3H) (Saito et al., 2013). In addition, the genes that involve in the late steps of anthocyanin biosynthesis encode enzymes including dihydroflavonol 4-reductase (DFR, *a1*) (Bernhardt et al., 1998), anthocyanidin synthase (ANS, *a2*) (Cheng et al., 2014), anthocyanidin 3-O-glucosyltransferase (AGT, *bz1*) (Ford et al., 1998; Fukuchi-Mizutani et al., 2003), anthocyanin 3-O-glucoside-6’’-O-malonyltransferase (MAT, *aat1*) (Paulsmeyer et al., 2017), and glutathione-S-transferase (GST, *bz2*) (Alfenito et al., 1998). The regulation of the anthocyanin biosynthetic pathway is directed by three families of transcriptional factors: *red1* (*r1*) and *booster1* (*b1*) which belong to the basic helix-loop-helix (bHLH) transcription factor family, *colorless1* (*c1*), *purple plant1* (*pl1*) and *pericarp color1* (*p1*) of the MYB-like transcription factors, and the WD40 factor *pale aleurone color1* (*PAC1*) (Sharma et al., 2011; Hu et al., 2016). The expression of each member of these families occurs in a tissue-specific manner during plant development. The pigmentation patterns of maize depend to a large extent on the allelic combination of the MYB and bHLH loci (Shen and Petolino, 2006).

Light is a crucial environmental factor that affects anthocyanin biosynthesis in many plants (He et al., 2016). For instance, strong light may significantly induce anthocyanin accumulation in grape peel and simultaneously induce the high expression of DFR, CHS, CHI, F3H, MT, F3’5’H, and GT (Azuma et al., 2015). Anthocyanin concentrations under blue and red light treatments were higher as compared with those in dark conditions in strawberries (Xu et al., 2014). In the grape ‘Marselan’, dark treatment can reduce anthocyanin content by changing the expression of anthocyanin synthase genes (*CHS*, *CHI*, *DFR*, *F3H*, *LDOX*, and *F3’5’H*) and regulatory genes (*MYB30*, *bHLH79*, and *bHLH121*) (Ma et al., 2019). The mechanism of light-induced anthocyanin synthesis in field maize has been examined in previous studies. Piazza et al. (2002) found members of the MYB-related *c1*/*pl1* gene family play important roles in the regulation of anthocyanin synthesis in maize in response to different light qualities. Plants with the *pl-bol3* allele, encoding an MYB-related transcription factor, showed a high level of anthocyanin accumulation in response to light (Piazza et al., 2002). In addition, light affects the distribution and packaging of anthocyanins within the vacuole, the morphology of anthocyanin-containing vacuolar structures, and the fusion of vacuolar inclusions (Irani and Grotewold, 2005). Although multiple light-responsive genes involved in anthocyanin synthesis have been well studied, the molecular mechanism of light intensity regulating anthocyanin synthesis in purple waxy maize kernels has not been reported yet to our knowledge. Therefore, studies on light regulation of anthocyanins are of great interest for the improvement of anthocyanin production in waxy maize kernels.

In this study, we investigated photosensitive waxy maize inbred line SWL502, which synthesized a significantly decreased amount of total anthocyanin with bagging, through a large number of bagging screenings. Here, we analyzed the transcriptomic and metabolomic data of bagged and unbagged waxy maize kernels and identified system-wide alterations in metabolites, structural genes, and regulation in response to light and dark treatments. This is the first in-depth look at how light affects the pigmentation of purple waxy maize kernels, and it paves the way for future studies and genetic manipulations of waxy maize kernel coloration.

## Materials and methods

### Plant materials and treatments

The purple waxy maize inbred line SWL502 was grown in the summer of 2021 at the experimental station of Shanghai Academy of Agricultural Sciences, Shanghai, China. After pollination, the whole ears were covered with foil paper bags for dark treatment (D) which were completely light‐impermeable, on June 16th, 2021. Meanwhile, unbagged ear samples, which were grown under natural light (L) conditions, served as controls. For each treatment, kernels from the middle part of three ears were sampled, mixed, and randomly divided into nine biological replicates: three for RNA-seq, and six for metabolome analysis. Kernel samples were collected at 12, 17, and 22 days after pollination (DAP), which were immediately frozen in liquid nitrogen and stored at -80°C for storage after sampling.

### Color phenotypic measurement

The color of the maize kernel was determined instrumentally by a Konica Minolta CM-5 colorimeter (Osaka, Japan) in the L* a* b* color space (CIELAB). Chroma, an expression of the purity or saturation of a single color, was calculated by using (a*^2^+b*^2^)^0.5^. Hue value measures the most obvious value of a color and was calculated by using arctan (b*/a*). The color index of red grapes (CIRG) was calculated with the formula CIRG = (180-*h*)/(*L*^∗^ + *C*) (Carreño et al., 1995). Saturation = Chroma/L*. The top ten kernels from random positions on each ear were used for colorimeter recording. The colorimeter was calibrated using a white and black reference before measurements and calibrated every 15 minutes during measurements. Each sample was analyzed three times.

### Extraction and assessment of anthocyanins contents

Anthocyanin mixtures from the waxy maize kernels were extracted and quantified according to the agricultural standard of China (NY/T 2640-2014) with modification. In brief, frozen waxy maize kernels were ground into powder in liquid nitrogen. Approximately 1 g of the maize powder was added in 10 mL of the ethanol-water-HCl mixture (2:1:1, v:v:v) for ultrasonic extraction for 30 minutes, boiling water bath for 1 h, and followed by centrifugation to collect the supernatant. The supernatant was then passed through a 0.45 μm membrane filter (ANPEL, China), and analyzed by ultra-performance liquid chromatography (UPLC) using a BEH C18 analytical column (2.1 mm × 100 mm, 1.7 μm, Waters, USA). A quantitative analysis of anthocyanin compounds was performed by an Acquity I-Class UPLC system with an ultraviolet detector (Waters, USA) using a formic acid-acetonitrile mixture (1:99, v:v) and formic acid-acetonitrile mixture (1:99, v:v) of the following gradient: 0 min, 10% A; 5 min, 18% A; 6 min, 18% A; 8 min, 10% A. The flow rate was 0.4 ml min^-1^ at 40 °C. The wavelength was set at 530 nm. Delphinidin (CAS: 528-53-0), cyanidin (CAS: 528-58-5), petunidin chloride (CAS: 1429-30-7), pelargonidin (CAS: 134-04-3), peonidin (CAS: 134-01-0), and malvidin (CAS: 643-84-5) were used as standards for anthocyanins. All samples were analyzed in six biological triplicates.

### Transcriptome sequencing and data analysis

Total RNA was extracted using the TRIzol reagent (Invitrogen, CA, USA) according to the manufacturer’s protocol. RNA purity and quantification were evaluated using the NanoDrop 2000 spectrophotometer (Thermo Scientific, USA). RNA integrity was assessed using the Agilent 2100 Bioanalyzer (Agilent Technologies, USA). Then the libraries were constructed using VAHTS Universal V6 RNA-seq Library Prep Kit (Vazyme Biotech Co., Ltd., China) according to the manufacturer’s instructions. The libraries were sequenced by an Illumina Novaseq 6000 platform in OE Biotech Co., Ltd. (Shanghai, China).

Raw reads were processed using FASTQC and Trimmomatic (Bolger et al., 2014) to obtain clean reads. Then the clean reads for each sample were mapped to the B73_RefGen_v4 genome using HISAT2 (Kim et al., 2015). FPKM (fragments per kilobase of transcript per million fragments mapped) of each gene was calculated and the read counts of each gene were obtained by HTSeq-count (Anders et al., 2015). Principal component analysis (PCA) was performed by the gmodels in R v 3.2.0 (Ihaka and Gentleman, 1996). Genes with a false discovery rate (FDR) < 0.05, and absolute fold change (FC) > 1.5 were selected, and the differentially expressed genes (DEGs) analysis was performed in the R package of DESeq2 (v1.30.1). The functions of these DEGs were analyzed by enrichment analysis using the R package ClusterProfiler (v3.18.1). The heatmap was generated in the pheatmap package (v1.0.12).

### Metabolite extraction and separation by liquid chromatography-mass spectrometry (LC-MS)

A total of 80 mg of freeze-dried maize kernels were mixed with 800 μl of methanol-water (7:3, v:v) and crushed for 2 min at 60 Hz using a mixer mill (Wonbio Biotechnology CO., LTD, China), followed by ultrasonic extraction in an ice-water bath for 30 min, and extracted overnight at -20°C. After centrifugation at 13000 rpm and 4°C for 10 min, the supernatant was filtered using a 0.22 μm organic phase pinhole filter. The filtrate was then transferred to an LC injection vial and stored at -80°C until LC-MS analysis. Using a UHPLC-ESI-MS/MS system, the sample extracts were examined (UHPLC, Dionex U3000 UHPLC; MS, QE plus). The analysis was conducted using an ACQUITY UPLC HSS T3 column (100 mm×2.1 mm, 1.8 μm). The mobile phase consisted of solvent A (0.1% formic acid in water) and solvent B (0.1% formic acid in acetonitrile). Using a gradient program of 95% A and 5% B as the initial condition, sample measurements were conducted. Within 16 minutes, a linear gradient of 5% A and 95% B was programmed, which was maintained for 1 min. The flow rate was maintained at 0.35 mL·min^-1^ throughout the course. The column oven was adjusted to 45°C, and the injection volume was 2 μL. Data were collected in both positive and negative ion modes.

### Metabolite extraction and separation by gas chromatography-mass spectrometry (GC-MS)

A total of 80 mg of maize kernels were placed in a 1.5 mL tube with 20 μL of internal standard (Lmur2-chloro-phenylalanine, 0.06 mg·mL^-1^, methanol as the dissolving agent). The tube was added with 360 μL of precooled methanol and put in the refrigerator at -20°C for 5 min. After grinding (60 Hz, 2 min) with steel balls, the powder was extracted by ultrasonic for 30 min at 0°C. Then 200 μL of chloroform and 400 μL of water were added to the powder and mixed evenly. After ultrasonic extraction in ice water for 30 min, the solution was placed at -20°C for another 30 min, followed by centrifugation for 10 min at 13 000 rpm at 4°C. Afterwards, 150 μL of the supernatant was transferred into a glass vial and dried by a centrifugal dryer. The obtained dried powder was mixed with 80 μL of methoxyamine hydrochloride pyridine solution (15 mg·mL^-1^). After vortex shaking for 2 min, the oximation reaction was carried out in a 37°C shaking incubator for 90 min. The sample was then added with 50 μL of BSTFA (including 1%TMCS) as the derivatization reagent, 20 μL of n-hexane, and 10 μL of ten types of internal standards (C8/C9/C10/C12/C14/C16/C18/C20/C22/C24, all chloroform configuration). After vortex shock for 2 min, the reaction was set for 60 min at 70°C. Finally, GC-MS analysis was performed at room temperature for 30 min. The obtained sample extracts were examined using DB-5MS column (30 m × 0.25mm × 0.25 μm, Agilent J&W Scientific, Folsom, CA, USA) with parameters set as follows: carrier gas, high purity helium; flow rate, 1.0 mL·min^-1^; and injection port temperature, 260°C. The injection volume was 1 μL, and the solvent delay was 5 min without shunt injection. Programmed heating was set as follows: the initial temperature of the column incubator was 60°C, holding for 0.5 min, then with a heating rate of 8°C min^-1^ to 125°C, 8°C min^-1^ to 210°C, 15°C min^-1^ to 270°C, 20°C min^-1^ to 305°C, maintaining for 5 min. Mass conditions were as follows: electron impact (EI); full scan mode with a range of 50-500 m; ion source temperature at 230°C; MS quard temperature at 150°C; electron energy at 70 eV. Quality control samples were prepared by mixing equal volumes of the extracts of all samples.

### Metabolome data analysis

The original LC-MS data were processed by the software Progenesis QI V2.3 (Nonlinear, Dynamics, Newcastle, UK) for baseline filtering, peak identification, integration, retention time correction, peak alignment, and normalization to obtain the data matrix. The unsupervised PCA of LC-MS and GC-MS data after unit variance scaling was carried out by using the *prcomp* function in R. The package of Simca (v14.0) was used to extract the variable importance in the projection (VIP) values from the OPLS-DA results, including score graphs and permutation plots. To prevent overfitting, a permutation test was performed 200 times. Differential metabolites consisting of metabolites with VIP > 1 and absolute Log2FC >= 1 were chosen for further study. The annotated metabolites were linked to the KEGG database. Pathways with substantially upregulated metabolites were then screened in MetaboAnalyst (v3.0), and their significance was established using the *p* values of the hypergeometric test. The Mfuzz (v 2.56.0) package was used for all trend analyses.

### Quantitative real-time polymerase chain reaction (qRT-PCR) analysis

The qRT-PCR analysis was performed on an SYBR Green system (TaKaRa, Dalian, China). The reaction was carried out in qTOWER 2.2 Quantitative Real-Time PCR Thermal Cyclers (Germany). The cycling conditions were 3 min of predenaturation at 95°C, then 95°C for 10 s, 60°C for 30 s, and 72°C for 90 s (39 cycles). The housekeeping gene *Actin* was used as an internal control to calculate relative gene expression in this study. The gene expression for each sample was measured by qRT-PCR using the relative quantification method (Gao et al., 2022). Primers were designed using Premier 6 software (Premier Biosoft, United States) and were listed in **Supplementary Table 1**. The same procedure was performed across all three independent biological and technical repeats.

## Results

### Light-induced pigmentation drastically changed in the waxy maize inbred line of SWL502

**Figure 1:**
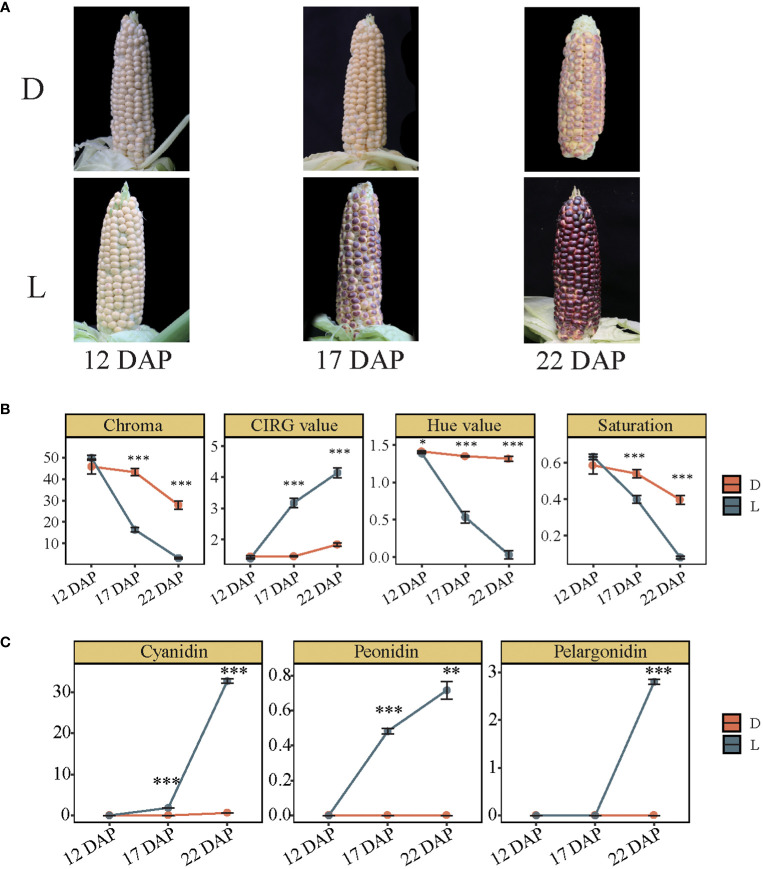
Pigmentation and anthocyanin content variation in purple waxy maize kernels between light and dark treatments. **(A)** Pigment changes of purple waxy maize kernels caused by natural light (L) and dark treatments by bagging (D). **(B)** Comparison of four color indexes including Chroma, CIRG value, Hue value, and Saturation. The error bar represents the standard error. **(C)** The contents of cyanidin, peonidin, and pelargonidin in purple waxy maize kernels in L and D. The error bar represents the standard error. The level of significance between D and L is indicated as * (P < 0.05), ** (*P* < 0.01), and *** (*P* < 0.001) based on Student’s t-test.

The effect of light on pigmentation in SWL502 kernel was investigated, and the findings revealed that there was no significant difference in kernel color between bagged and unbagged ears at 12 DAP. However, the color of kernels differed dramatically at 17 and 22 DAP, indicating that 12 to 22 DAP is a crucial period for pigment formation in SWL502 (**Figure 1A**). Subsequently, various color indices were quantitatively analyzed on the kernels treated in D and L conditions. Among them, the Chroma value decreased rapidly in L, with a significantly greater rate of decrease than that in D (**Figure 1B**). The Hue and Saturation values showed a similar trend to Chroma, indicating a rapid decrease in the Hue value and Chroma index in the corn kernels. In contrast, the CIRG value increased significantly in L but showed no significant change in D (**Figure 1B**). Further examination of the alterations of major anthocyanin precursors revealed that light significantly enhanced peonidin content from 12 to 17 DAP, whereas cyanidin and pelargonidin levels of this period remained unchanged. However, synthesis of the three anthocyanins (cyanidin, peonidin, and pelargonidin), was significantly induced by light from 17 to 22 DAP, indicating temporal variations in the accumulation of the three anthocyanins. The derivatives of peonidin may be the principal compounds of color formation at 17 DAP, whereas the derivatives of pelargonidin and cyanidin may become the principal compounds at 22 DAP in SWL502 (**Figure 1C**). All these results suggest that light-induced transcription regulates the metabolism of cyanidin, pelargonidin, and peonidin differentially.

### Dynamic changes in the transcriptome in purple waxy maize kernels induced by dark treatment

**Figure 2:**
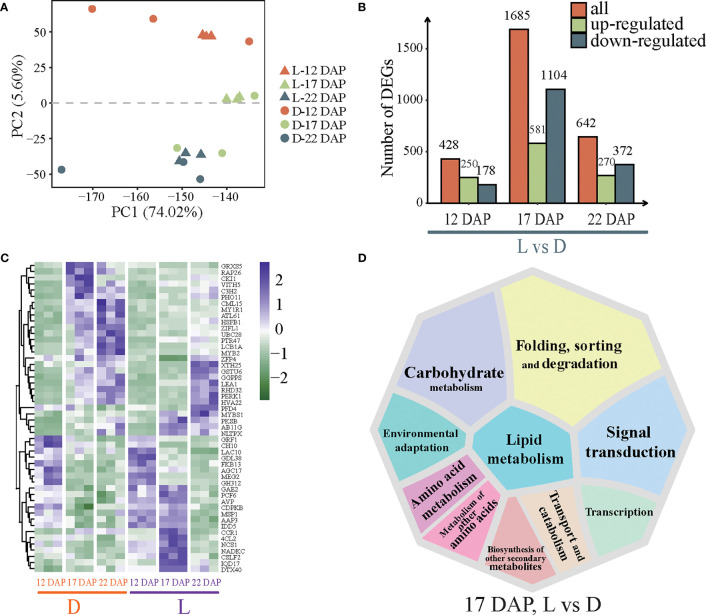
Transcriptome overview of the effects of light and dark treatments on purple waxy maize kernels. **(A)** Principal component analysis (PCA) of effects on the purple waxy maize kernel transcriptome in D and L, where time points are represented with colors, while treatments are represented with shapes. **(B)** Bar graph of differentially expressed genes (DEGs) between D and L. **(C)** Hierarchy clustering of the top 50 DEGs across samples. The row in the heatmap represents genes, and the column indicates samples. The color gradient, ranging from green to purple represents low and high values of gene expression. **(D)** The top ten enriched KEGG pathways of DEGs at 17 DAP.

DEGs were analyzed to investigate the underlying mechanisms of the differential accumulation of anthocyanins between the two treatments. PCA was used to measure the gene expression levels of each biological duplicate (**Figure 2A**). PC1 distinguished the 12 DAP and 17 DAP phases of maize kernel development effectively, whereas PC2 was more accurate in identifying 12 DAP, 17 DAP, and 22 DAP (**Figure 2A**). We thus assume that developmental stages had a strong impact on maize kernel transcriptome. The differential analysis identified 428 (250 up-regulated and 178 down-regulated), 1685 (581 up-regulated and 1104 down-regulated), and 642 (270 up-regulated and 372 down-regulated) DEGs at 12 DAP, 17 DAP, and 22 DAP in D conditions, respectively, compared with their corresponding L conditions (**Figure 2B**). The results indicated that the kernels at 17 DAP had the highest sensitivity to light. The *MYBS1*, *PKSB*, *AB11G*, and *NLTPX* genes were induced by light, while *GRXS5*, *MY1R1*, *C3H2*, *MYB2*, and *ZIFL1* were significantly induced by darkness (**Figure 2C**). KEGG enrichment analysis of DEGs at 17 DAP showed that they were mostly engaged in metabolic pathways such as carbohydrate metabolism, production of secondary metabolites, amino acid metabolism, lipid metabolism, signal transduction, and folding, sorting, and degradation, indicating that light had considerably influenced the metabolism of numerous organic macromolecules and the accumulation of secondary metabolites in maize kernels (**Figure 2D**). Overall, dark treatment led to the overexpression of numerous genes in maize kernels, indicating that developmental stages, other than dark treatment, had extensive effects on purple waxy maize kernels.

### Metabolite accumulation significantly altered under dark treatment

Using PCA, the degree of metabolite variation in each biological duplicate was evaluated (**Supplementary Figure 1**). PC1 efficiently separated the samples at the DAP-22 stage from those at other stages under light treatment conditions, indicating that the metabolites in the samples at the 22 DAP differed significantly from those at other stages under light treatment. A total of 2171 metabolites were identified from all the samples, of which 1962 were recognized as categorized compounds during screening. Differentially expressed metabolites (DEMs) consisting of metabolites with VIP>1 were chosen for further study. DAP17 and DAP22 in the light group had 257 and 292 DEMs compared with DAP12, respectively. And DAP17 and DAP22 in the dark group had 281 and 258 specific DEMs compared with DAP12, respectively (**Supplementary Figure 1**). These results showed that the metabolites at 22 DAP in L, including cellobiose, hexitol, 1,3-diaminopropane, and dihydrocholesterol, were substantially different from other groups (**Supplementary Figure 1**). The results of the KEGG enrichment analysis conducted between the D and L conditions at 22 DAP, demonstrated that DEMs between the D conditions were mostly engaged in flavonoid biosynthesis, cyanoamino acid metabolism, and phenylalanine metabolism (**Supplementary Figure 1**).

### Light and dark treatments resulted in differential transcriptional and metabolic changes in SWL502

**Figure 3:**
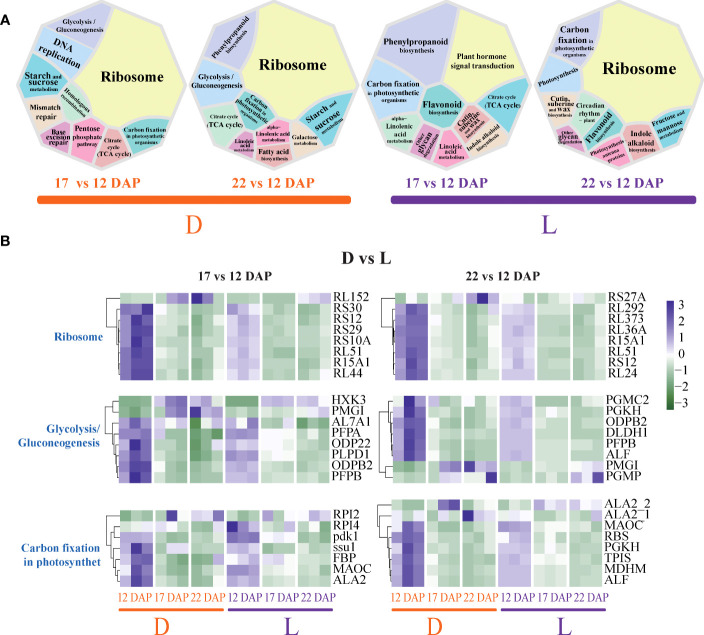
Differences in transcriptome pathways across developmental stages of purple waxy maize kernels under light and dark conditions. **(A)** Comparison of the DEGs-involved pathways. **(B)** Specific changes of crucial genes. The color gradient, ranging from green to purple represents low and high values of gene expression.

From the above analyses, we consider that 17 DAP and 22 DAP were key stages for the coloration of maize kernels. We investigated the variations in transcriptome and metabolome across maize kernel development stages, especially those between 17 DAP and 22 DAP under light and dark conditions. Ribosome, glycolysis/gluconeogenesis, starch, and sucrose metabolism, and the TCA cycle were identified as the main pathways of transcriptome changes at 17 DAP under the D condition (**Figure 3**). ABC transporters, starch and sucrose metabolism, and the TCA cycle were identified as the main metabolome components changed under the D condition (**Supplementary Figure 2**). Phenylpropanoid biosynthesis, flavonoid biosynthesis, and the TCA cycle were the main pathways of transcriptome alterations at 17 DAP in natural light conditions (**Figure 3**). Phenylpropanoid biosynthesis and the TCA cycle were the primary pathways of metabolome changes at 17 DAP under light treatment (**Supplementary Figure 2**). The translation-related ribosomal genes *RL152*, *RS10A*, *RS29*, etc. were down-regulated at 17 DAP with light, and the *PFPB*, *ODPB2*, and *PLPD1* genes related to glycolysis/gluconeogenesis showed a similar trend (**Figure 3**). This indicates that dark treatment increased translation levels and glucose metabolism in maize kernels. In addition, genes related to photosynthetic carbon fixation, such as *RPI4* and *pdk1*, were up-regulated at 17 DAP under light treatment (**Figure 3**), indicating that light at this stage may increase carbon fixation in maize kernels. Metabolites, such as 4−Hydroxycinnamic acid (4-HCA), chlorogenic acid, and L−valine, decreased at 17 DAP under light treatment (**Supplementary Figure 2**). Moreover, ribosome, phenylpropanoid biosynthesis, starch and sucrose metabolism, and TCA cycle were the main pathways of transcriptome alterations at 22 DAP in D (**Figure 3**), whereas ABC transporters, galactose metabolism, and the TCA cycle were the primary pathways of metabolome modifications (**Supplementary Figure 2**). Ribosome, cutin, suberine, and wax biosynthesis, fructose, and mannose metabolism, and photosynthesis were the primary pathways of transcriptome changes at 22 DAP in L (**Figure 3**). The primary pathways of metabolite changes were starch and sucrose metabolism, carbon fixation in photosynthetic organisms, and fructose and mannose metabolism at 22 DAP in L (**Supplementary Figure 2**). These findings imply that the transcriptome and metabolome display consistency in developmental stage alterations. Light also enhanced the expression of genes involved in the phenylpropanoid and flavonoid biosynthesis pathways at 17 DAP stage, and induced the expression level of genes involved in carbon fixation, cutin, suberine, and wax biosynthesis at the 22 DAP stage.

The translation-related ribosomal genes of *RL51*, *RS12*, *RL24*, etc., were down-regulated at 22 DAP in the L condition, and the *PGKH*, *PGMC2*, and *ODPB2* genes related to glycolysis/gluconeogenesis showed the same trend (**Figure 3**). In addition, genes related to photosynthetic carbon fixation, such as *RBS*, *MAOC*, and *MDHM*, also showed down-regulation at 22 DAP in L, which was different from that at 17 DAP (**Figure 3**). In L, the metabolites, such as mannose 6-phosphate (M6-P), D-fructose-6-phosphate (D-F6P), and glucose-6-phosphate (G6P), increased at 22 DAP (**Supplementary Figure 2**). All these results suggested that light and dark treatments differentially affect the key genes that are involved in translation-related functions, glucose metabolism, and flavonoid metabolism pathways during purple waxy maize kernel pigmentation development.

### Gene alterations in the anthocyanin biosynthesis pathway

**Figure 4:**
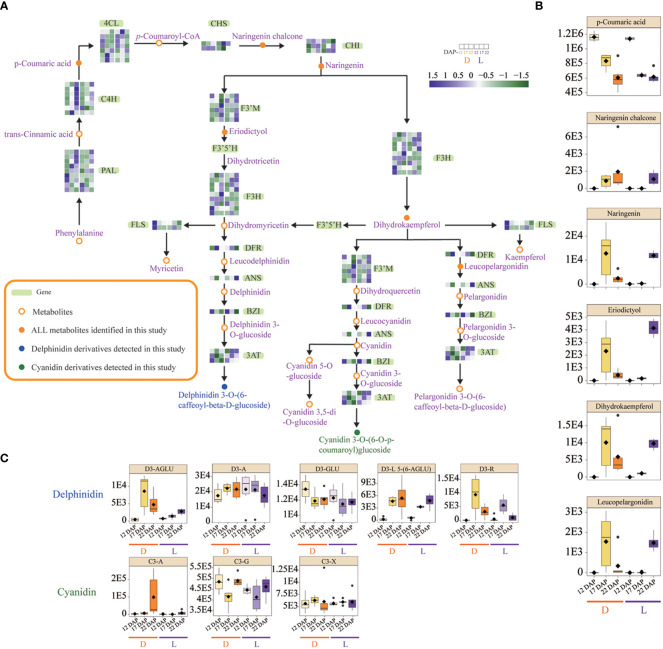
Reconstitution of anthocyanin biosynthetic pathway in purple waxy maize kernels, and expression changes of structural genes and metabolites in L and D. **(A)** Expression changes of structural genes in the anthocyanin biosynthetic pathway. The code genes represented by gene symbols are described as follows: PAL, phenylalanine ammonia lyase; C4H, cinnamate-4-hydroxylase; 4CL, 4-coumarate-CoA ligase; CHS, chalcone synthase; CHI, chalcone isomerase; F3'M, flavonoid 3′-monooxygenase; F3H, flavanone 3-hydroxylase; F3′5′H, flavonoid 3′5′ hydroxylase; FLS, flavonol synthase; DFR, dihydroflavonoid reductase; ANS, anthocyanidin synthase; BZI, bZIP transcription factor. The color scale reflected the log-transformed FPKM values. **(B)** Changes in the contents of metabolites involved in the anthocyanin biosynthesis pathway. **(C)** Changes in the contents of derivatives of delphinidin and cyanidin the two anthocyanin precursors. D3-AGLU, delphinidin 3-(acetylglucoside); D3-A, delphinidin 3-arabinoside; D3-GLU, delphinidin 3-glucoside; D3-L 5-(6-AGLU), delphinidin 3-lathyroside 5-(6-acetylglucoside); D3-R, delphinidin 3-rutinoside; C3-A, cyanidin 3-arabinoside; C3-G, cyanidin 3-gentiobioside; C3-X, cyanidin 3-xyloside.

Expression profiling of structural genes involved in anthocyanin biosynthesis was analyzed to evaluate their functions in the pathway (**Figure 4**). Three *4CL* genes were induced at 12 DAP in L, whereas their expressions were down-regulated at 17 DAP in D. In L, one *CHS* gene, two *F3H* genes, two *3AT* genes, and one *ANS* gene were simultaneously induced at 17 DAP. The results showed that the expression of the *DFR* gene was unaffected in both L and D. However, the expression of five *F3’M* genes was dramatically up-regulated at 22 DAP in L (**Figure 4**). *F3’M* catalyzes the production of dihydroquercetin from dihydrokaempferol, resulting in the accumulation of cyanidin derivatives, which may be the predominant anthocyanin types in SWL502. At 22 DAP, light enhanced the accumulation of metabolites, such as eriodictyol, naringenin, and dihydrokaempferol, which may also be associated with kernel pigmentation (**Figure 4**). In addition, eight derivative metabolites of delphinidin and cyanidin were identified. Cyanidin 3-gentiobioside (C3-G) was the most significant anthocyanin type in SWL502 (**Figure 4**).

As anthocyanins are flavonoid derivatives, flavonoid metabolites in L and D were also investigated. Comparing L with D, 13 flavonoids accumulated differently (**Supplementary Figure 3**). Integrin and kaempferol were the predominant flavonoid types in SWL502, and both of them were dramatically up-regulated by light at 22 DAP. The concentration of flavonoids may also cause major changes in maize kernel color (**Supplementary Figure 3**). These findings suggest that the genes *4CL, CHS, F3H, ANS*, and *F3’M* were significantly influenced by light, with the notable exception of *PAL*, *DFR*, etc. The light-induced *F3’M* gene expression drove the biosynthesis of anthocyanins in purple maize kernels in the form of cyanidin, which may also lead to a significant accumulation of cyanidin 3-gentiobioside.

### Light affected transcription factor (TF) expression and hormone synthesis

**Figure 5:**
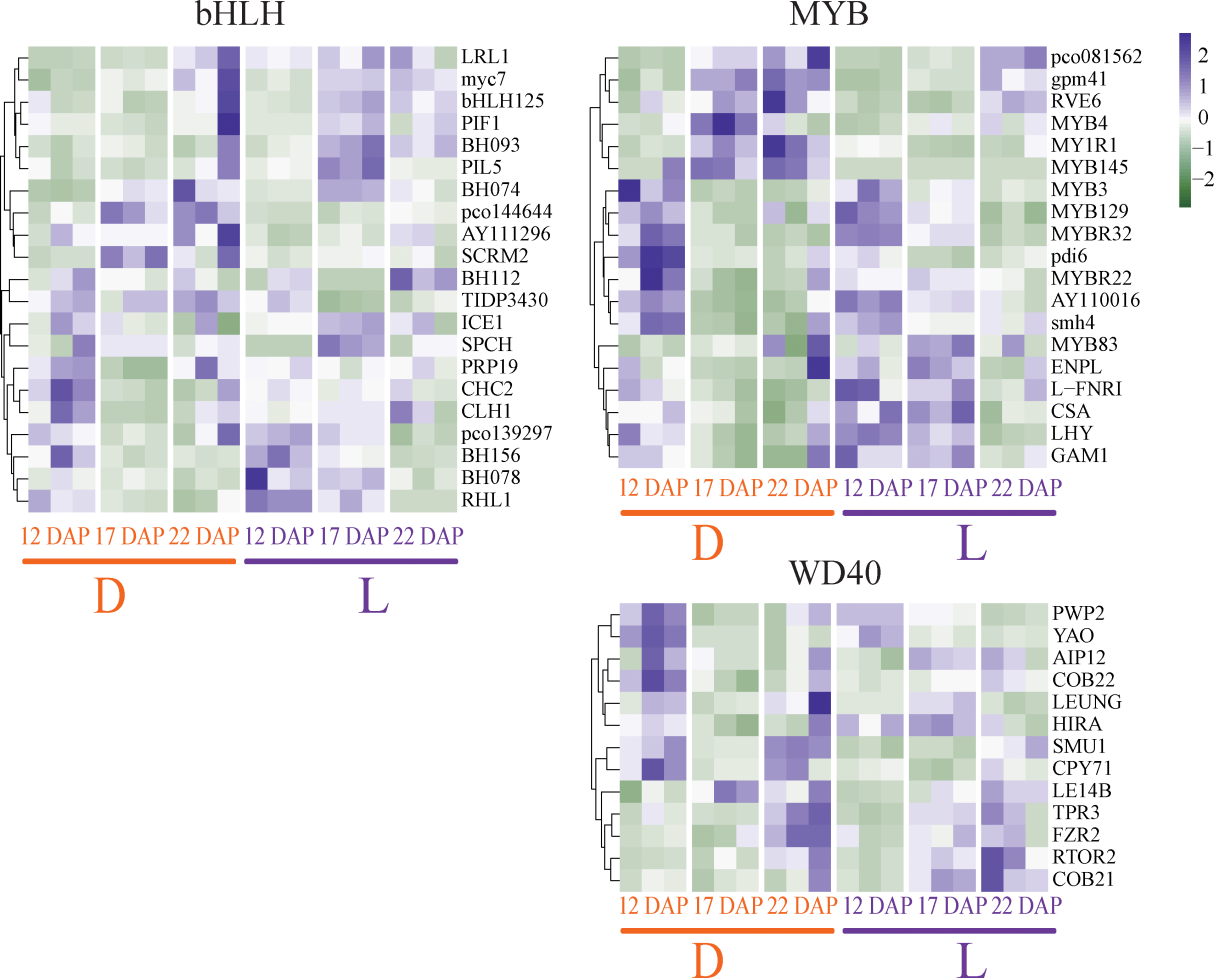
Expression changes of transcription factors (TFs) regulating anthocyanin biosynthesis pathway in purple waxy maize kernels, including the bHLH, MYB, and WD40 TF family members. The color scale reflected the log-transformed FPKM values.

Light and dark treatments may impact the regulation of the MYB-bHLH-WDR (MBW) transcription complex, which is a crucial transcriptional regulator in the flavonoid pathway in the plant. Overall, 21 bHLH, 18 MYB, and 13 WD40 TFs were differentially expressed between light and dark conditions. The expressions of seven *bHLH* TFs were up-regulated by light induction, including *LRL1, myc7, bHLH125, PIF1, BH093, PIL5, and BH074* (**Figure 5**). The expression of twelve MYB TFs, including *MYB129, MYBR32, pdi6, MYBR22, AY110016, smh4, MYB83, ENPL, LFNRI, CSA, LHY, and GAM1*, were also induced by light treatment. Light also dramatically up-regulated WD40 TFs, including TPR3, FZR2, RTOR2, and COB21 (**Figure 5**). However, the timing of the expression of the three MBW TFs did not coincide. Under light treatment, expressions of *bHLH* and *WD40* were mostly induced at 17 and 22 DAP, while that of *MYB* was primarily induced at 12 and 17 DAP. This result implies that MYB is the first TF to be activated by light, and 17 DAP may be critical for the MBW complex to perform light-driven transcriptional control. Furthermore, anthocyanin synthase-related TFs were verified by qRT-PCR, showing consistent results with RNA-seq (**Supplementary Figure 4**).

In addition, seven TF families (NAC, bZIP, Dof, MIKC-MADS, HD-ZIP, C2H2, and ERF) were also identified as important regulators involved in light response (**Supplementary Figure 5**). Among them, the TF genes in NAC, MIKC-MADS, and HD-ZIP may play a negative regulatory role, as most of them were down-regulated in L, but up-regulated in D. In contrast, the bZIP, Dof, C2H2, and ERF family members may play opposite regulatory roles, as most of them are up-regulated by light (**Supplementary Figure 5**). Altogether, besides the MBW complex, members of other TFs may also play potential light-induced regulatory roles in purple waxy maize kernels.

### Short time-series analyses of DEGs and DEMs revealed light- and dark-specific modules

**Figure 6:**
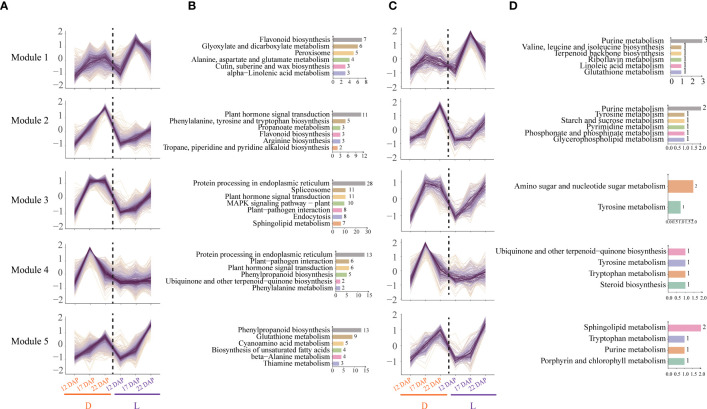
Time-series analysis and KEGG pathway enrichment analysis of the differentially expressed genes (DEGs) and differentially expressed metabolites (DEMs). **(A)** Trend analysis of DEGs. The abscissa represents the time points and treatments, and the ordinate represents the change factor of genes. **(B)** KEGG enrichment analysis of DEGs. **(C)** Trend analysis of DEMs. The abscissa represents the time points and treatments, and the ordinate represents the change factor of metabolites. **(D)** KEGG enrichment analysis of DEMs.

Short time-series analyses of total DEGs and DEMs were conducted to comprehend the response of purple waxy maize kernels to L and D treatments. All DEGs and DEMs were grouped into 12 clusters, 5 of which with comparable transcriptome and metabolome variation patterns were filtered out. Module 1 is a particular increase in gene expression and metabolites at 17 DAP in L (**Figure 6A**). Functional enrichment analysis revealed that the genes in Module 1 were primarily involved in the biosynthesis of flavonoid, brassinosteroid, benzoxazinoid, cutin, suberine, and wax, as well as the metabolism of glyoxylate and dicarboxylate (**Figure 6**). Metabolites in Module 1 were primarily involved in purine metabolism and the biosynthesis of terpenoid backbone, valine, leucine, and isoleucine (**Figure 6**). These results suggest that the L group of 17 DAP focused on the metabolism of flavonoid, cutin and wax, terpenoid, purine, and amino acid in purple waxy maize kernels. Module 2 had a particular increase in gene expression and metabolites at 22 DAP in L (**Figure 6A**), including Module 2 of both the transcriptome and metabolome. The genes and metabolites in Module 2 were mostly connected with the production of hormones, phenylalanine, tyrosine, and tryptophan biosynthesis, indicating that dark treatment induced substantial variations in hormone-related amino acids and pigment synthesis in waxy maize kernels (**Figure 6B**). The genes and metabolites of Module 3 showed continuously high expression at 17 and 22 DAP in D. KEGG functional enrichment analysis revealed that the genes in Module 3 were mostly engaged in the MAPK signaling pathway, plant-pathogen interaction, and other pathways, whilst the metabolites in Module 3 were predominantly involved in tyrosine metabolism (**Figure 6B**). Module 4 has a specific high expression of genes and metabolites in D at 17 DAP (**Figure 6A**). A few of the genes and metabolites of Module 4 involved in the pathways were identical to those in Module 3 (**Figure 6B**). These results showed that dark treatment induced a defensive response in plants by activating pathways associated with biotic stress. Module 5 demonstrated a continuous increase in the expressions of genes and metabolites from 12 to 22 DAP in L (**Figure 6A**). KEGG enrichment analysis revealed that the genes in Module 5 are mostly engaged in phenylpropanoid biosynthesis, glutathione metabolism, and other pathways, while the metabolites in Module 5 are predominantly involved in the metabolism of sphingolipid, tryptophan, and purine (**Figure 6B**). These results demonstrated that light increased the expression of genes involved in the neutralization of starch buildup, cell wall formation, and antioxidant chemical synthesis in purple waxy maize kernels.

## Discussion

Due to its high flavonoid content and potential health-enhancing effects, purple maize is an emerging star in the ingredient market (Elisa et al., 2021; Salvador-Reyes et al., 2022). Numerous investigations have shown that chemicals derived from purple maize have powerful antioxidant, anti-inflammatory, antimutagenic, anticancer, and antiangiogenic capacities (Rouf Shah et al., 2016; Lao et al., 2017; Coloma et al., 2019). In this study, the anthocyanin glycosides in SWL502 are cyanidin 3-gentiobioside, cyanidin 3-xyloside, cyanidin 3-arabinoside, delphinidin 3-arabinoside, delphinidin 3-(acetyl glucoside), delphinidin 3-glucoside, and delphinidin 3-rutinoside. Cyanidin 3-gentiobioside was found to be the most significant anthocyanin glycoside in purple waxy maize kernels, which is also a primary component of anthocyanins in black rice (Asem et al., 2015; Hao et al., 2015). Light-treated purple waxy maize kernels contain a high concentration of the pharmacologically active compound Kaempferol, which has antioxidant, anti-inflammatory, antibacterial, anticancer, cardioprotective, neuroprotective, antidiabetic, antiosteoporotic, antiestrogenic, anti-anxiety, analgesic, and anti-allergic properties (Chen and Chen, 2013; Kashyap et al., 2017; Jan et al., 2022). This suggests that light is an environmental stimulus for the biosynthesis of flavonoids and anthocyanins in purple waxy maize kernels. However, dark treatment suppressed the anthocyanin synthesis in purple waxy maize kernels.

Rapid production of anthocyanins occurs under light circumstances. Anthocyanin synthesis is regulated by light (Pech et al., 2022). Light induces the synthesis and accumulation of anthocyanins in purple maize seedlings (Shimakawa and Miyake, 2021). Under light treatment, peonidin content accumulated dramatically at 17 DAP, whereas pelargonidin and cyanidin concentrated significantly at 22 DAP. This demonstrates timing disparities between the accumulations of the three anthocyanins. The derivatives of peonidin may be the predominant anthocyanins during the early growth of purple waxy maize kernels, while those of pelargonidin and cyanidin prevailed in their later development stage. The pattern of anthocyanin buildup in purple waxy maize kernels is similar to that in several crops and vegetables. The anthocyanin content in purple heading Chinese cabbage increased gradually from the early stage of corm formation, and accumulated continuously during the whole plant development (He et al., 2020). Natural variations in the type, content and tissue-specific pattern of anthocyanins were observed in *Solanaceae* fruits and vegetables. Wild tomatoes in particular, may produce purple anthocyanin spots in strong light (Li et al., 2022).

Light not only affects the synthesis of anthocyanins, but also the synthesis of cutin and wax, and the maturation of purple maize (Silva et al., 1999; Giovannoni, 2001; Li et al., 2007; Lee and Suh, 2013). In this study, the transcriptome of purple waxy maize kernels was altered seriously by light at 17 DAP. The expressions of structural and regulatory genes in pathways including the biosynthesis of flavonoid, cutin, suberine, wax, brassinosteroid, and benzoxazinoid were induced by light during this stage. The buildup of chemicals like cutin and wax may hasten the hardening and keratinization of maize kernels. At 22 DAP, light promoted the expression of genes involved in starch metabolism and carbon fixation. In addition to accelerating coloration in purple maize, we hypothesize that light may also influence the accumulation and hardening of starch in kernels.

The anthocyanin pigmentation of purple maize requires the participation of many genes in the anthocyanin biosynthesis pathway (Chaves-Silva et al., 2018; Anirban et al., 2023), which has been recongnized with the discovery of potential key regulatory genes (Banerjee et al., 2023). Transcriptome analysis of purple corn 963 at different developmental stages showed that the expression of *CHI*, *CHS*, *LDOX* and *F3H* genes were the highest at 34DAP (Ming et al., 2021). A collection of TFs, including R2R3-MYB, bHLH, WD40, and members of numerous additional TF families, involved in the light-induced anthocyanin biosynthesis at the transcriptional level (Li, 2014; Pathak et al., 2022; Xie et al., 2022). For instance, MYBs played crucial roles in the light signals transduction in turnip (Wang et al., 2012). The BrTT8, interacting with BrPAP1 and BrTTG1, regulated the expression of LBGs (BrDFR, BrANS1, BrANS2, and BrUFGT) in response to light, hence playing a favorable role in anthocyanin biosynthesis in turnip (Yang et al., 2017). Here we identified 54 members of the R2R3-MYB, bHLH, and WD40 TF families that reacted differentially to light and dark, including 21 bHLH, 18 MYB, and 13 WD40 TFs. COB21 was identified as one of the 23 R2R3-MYB, bHLH, and WD40 positive regulators that are involved in the regulation of anthocyanins biosynthesis. The timing of expressions differed among the three TFs of MYB, bHLH, and WD. Under light treatment, bHLH and WD40 were mostly induced from 17 to 22 DAP, while MYB was primarily induced from 12 to 17 DAP. We assumed that MYB is the first TF to be induced by light, and the 17 DAP may be the critical period for the MBW complex to exert light-induced transcriptional regulation. Moreover, MYB is the promoter protein of the MBW transcriptional regulatory complex in purple waxy maize kernels, and MYB regulates anthocyanin production by binding bHLH and WD40.

Besides the positive regulators, we also identified possible negative regulators induced by darkness. For instance, MYB4, MYB145, MY1R1, and RVE6, which are mostly TFs from the MYB family, were supposed to be negative regulators in this study. In a previous study, the absence of BoMYBL2-1, an MYB family TF, led to the development of a purple hue in cabbages (Song et al., 2018). Intense light suppressed MYBL2 expression, resulting in a substantial buildup of anthocyanins in Arabidopsis (Dubos et al., 2008). We hypothesize that MYB4, MYB145, and MY1R1 may function as transcriptional repressors to inhibit anthocyanin production and their expression may be adversely controlled by light. Our research provided a useful model for investigating light induced pigmentation in cereals. Breeding anthocyanin-rich varieties is an important avenue to improve the quality of maize. This study revealed important regulatory and structural genes involved in anthocyanin biosynthesis pathway, which can be used in molecular breeding of maize. The combination of recombinant DNA technology and plant metabolic engineering will be a promising strategy to delelop maize lines with high anthocyanin content. The application of molecular marker-assisted selection is particularly useful in improving the nutritional values of maize, and is expected to cultivate breeding lines with higher anthocyanin content and quality (Cai et al., 2023).

## Conclusion

Light is a critical environmental factor that controls anthocyanin synthesis in purple waxy maize kernels. Purple corn growing under light treatment contains a high concentration of the pharmacologically active compound kaempferol, which has numerous health-enhancing properties. TFs R2R3-MYB, bHLH, and WD40 were involved in the regulation of anthocyanin biosynthesis. The expressions of these TFs were found to be differentially regulated by light and dark treatments, among which MYB plays a crucial role in the light-induced transcriptional regulation of anthocyanin biosynthesis. In addition, light promotes the synthesis of cutin and wax, major components for the hardening and keratinization of maize kernels. Overall, this study contributes to the understanding of the mechanism of light-induced accumulation of flavonoids and anthocyanins in purple waxy maize kernels and may have implications for the development of new functional foods and nutraceuticals.

## Data availability statement

The datasets presented in this study can be found in online repositories. The names of the repository/repositories and accession number(s) can be found in https://www.ncbi.nlm.nih.gov/sra, PRJNA952737.

## Author contributions

HZ and YL conceived the project and designed the experiments. YY, YFX, AK, CY, CW, and CZ performed the experiment and YL wrote the manuscript. Validation was done by WG, HW, YH, PS, and YG. Formal analysis was performed by WS, BB, and BS. The writing—review, and editing were done by XZ, YBX, and PB. All authors contributed to the article and approved the submitted version.
