# Supplementary material for: Integrative transcriptome and metabolome analysis reveals the mechanisms of light-induced pigmentation in purple waxy maize
Source: Front Plant Sci. 2023 Aug 15;14:1203284. doi: 10.3389/fpls.2023.1203284 (PMC10465178; doi:10.3389/fpls.2023.1203284)
Supplement: Supplementary file 1 [file DataSheet_1.docx]

**Supplemental table legends**

Supplementary Table 1 Primer sequence for qRT-PCR

**Supplemental figure legends**

Supplementary **Figure 1** Metabolome overview of the effects of light and darkness on purple waxy maize kernels. **(A)** Principal component analysis (PCA) of the effects on the purple waxy maize kernel metabolome under light (L) and dark (D) treatments, where colors represent developmental stages, and shapes represent treatments. **(B)** Venn diagram of differentially expressed metabolites (DEMs). **(C)** Hierarchical clustering analysis of the top 25 DEMs demonstrated the differential reaction in D and L. The color gradient, ranging from green to purple represents low and high values of metabolite expression. **(D)** The top ten enriched KEGG pathways of DEMs in the purple waxy maize kernels at 22 DAP.

Supplementary **Figure 2** Differences in metabolome pathways across developmental stages of purple waxy maize kernels under light and dark conditions. **(A)** Comparison of the differentially expressed metabolites (DEMs) involved pathways. **(B)** Specific changes of DEMs. 2-KIAA, 2-ketoisovaleric acid; 4-HCA, 4-hydroxycinnamic acid; D-F6P, D-fructose-6-phosphate; D-SP7-P, D-Sedoheptulose 7-phosphate; G6P, glucose-6-phosphate; L 7-O-beta-D-GLU, luteolin 7-O-[beta-D-glucuronosyl-(1->2)-beta-D-glucuronide]; M6-P, mannose 6-phosphate.

Supplementary **Figure 3** Effects of light and dark treatments on flavonoid metabolites in purple waxy maize kernels.

Supplementary **Figure 4** The qRT-PCR results confirm the transcriptome expression of RNA-seq. The error bar represents the standard error. The significant difference is indicated by * (*P* < 0.05), ** (*P* < 0.01), and *** (*P* < 0.001) based on Student’s t-test.

Supplementary **Figure 5** Other differentially expressed transcription factor (TF) genes affected by light and dark treatments. The color scale reflected the log-transformed FPKM values.
