# Supplementary figures and images for: Integrative transcriptome and metabolome analysis reveals the mechanisms of light-induced pigmentation in purple waxy maize
Source: Front Plant Sci. 2023 Aug 15;14:1203284. doi: 10.3389/fpls.2023.1203284 (PMC10465178; doi:10.3389/fpls.2023.1203284)

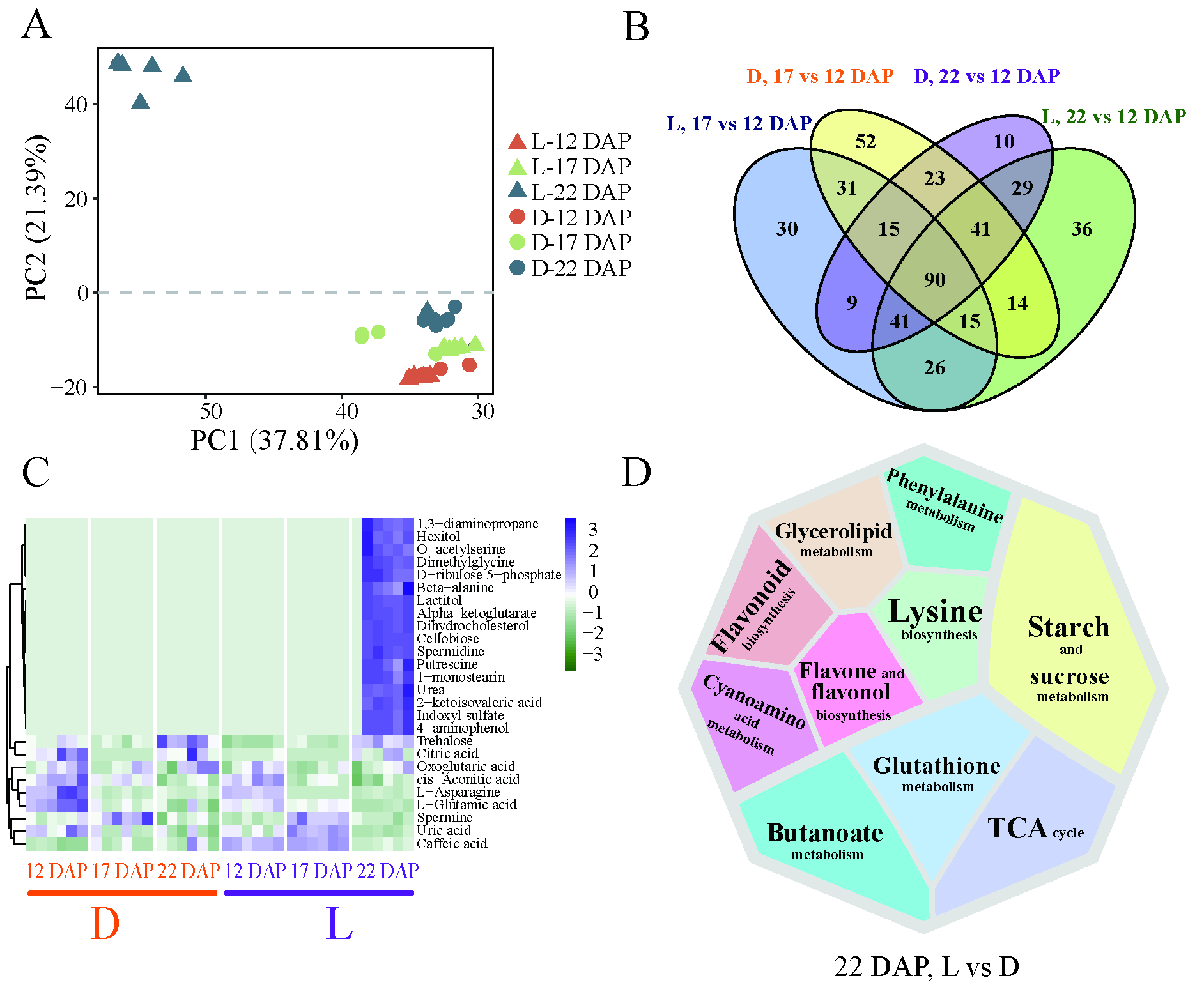

Supplement: Supplementary file 2 [file Image_1.tif]

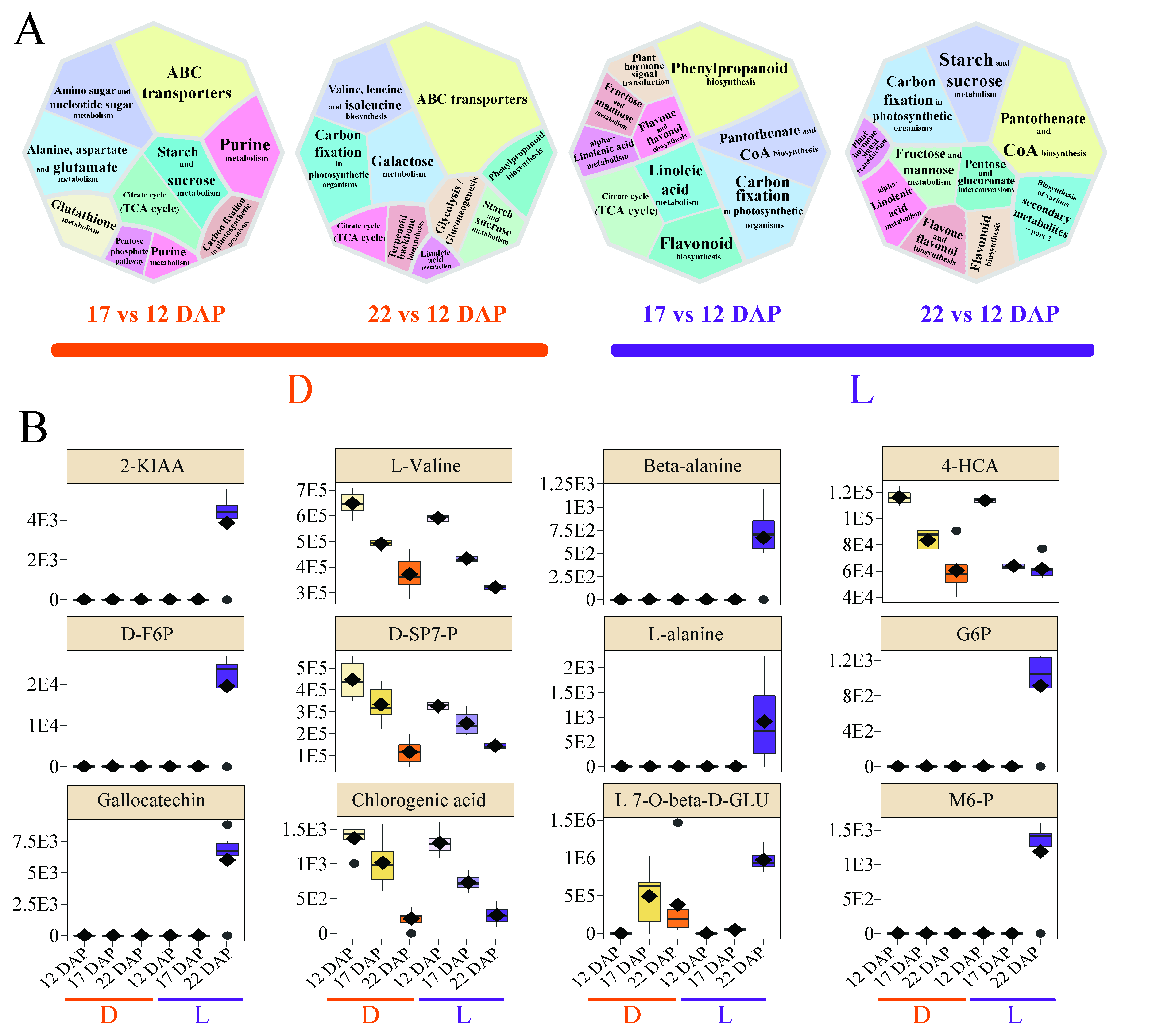

Supplement: Supplementary file 3 [file Image_2.tif]

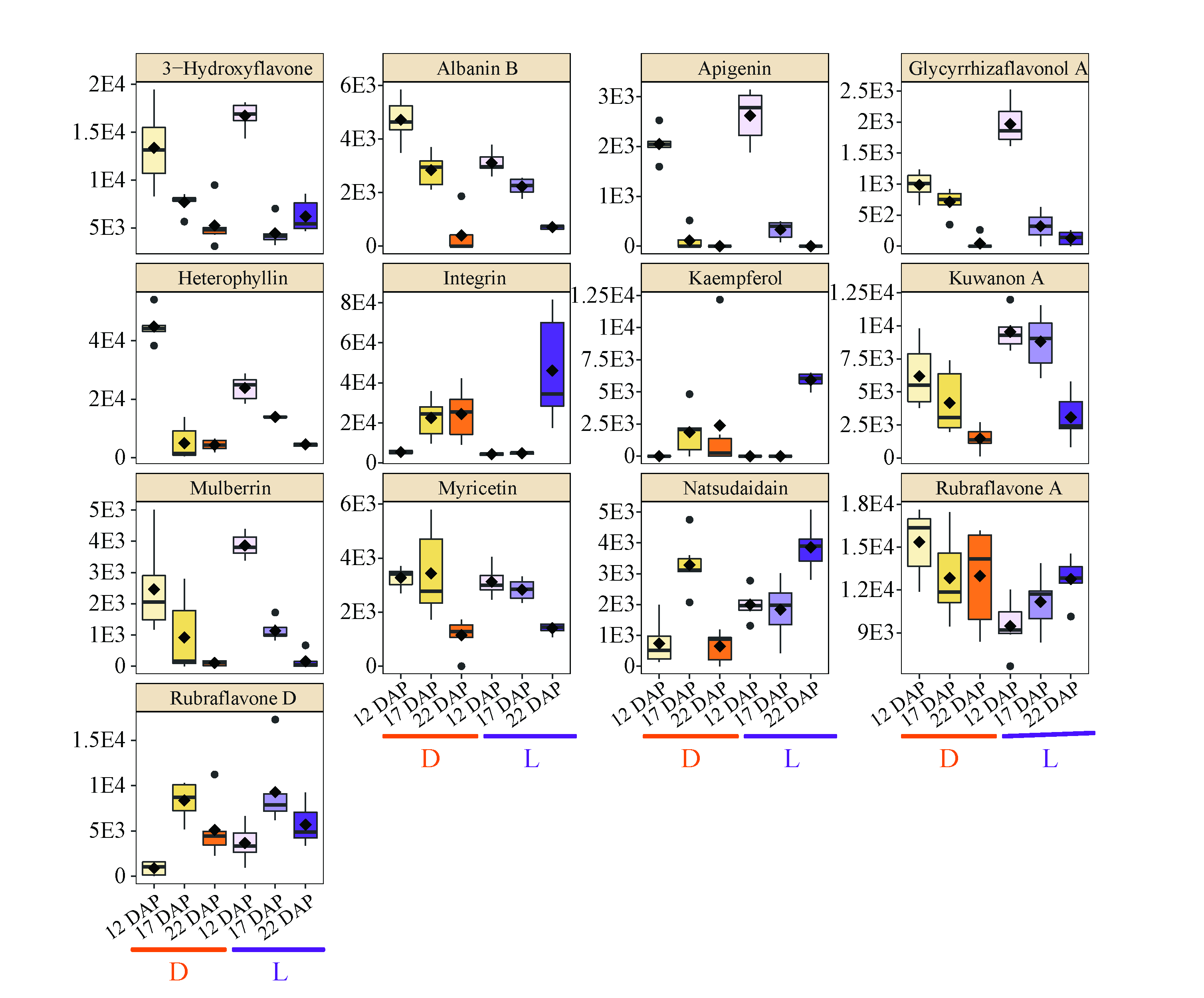

Supplement: Supplementary file 4 [file Image_3.tif]

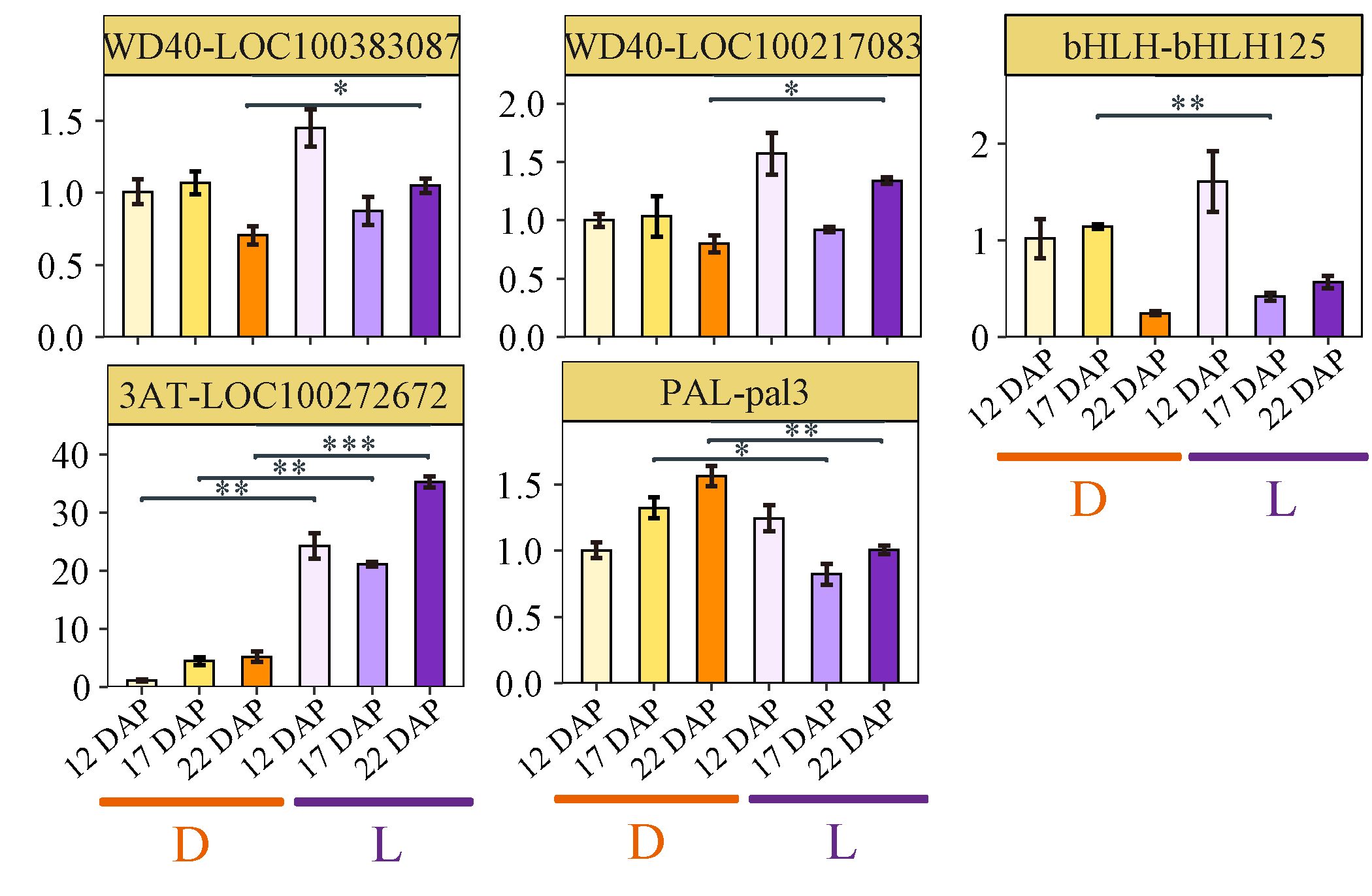

Supplement: Supplementary file 5 [file Image_4.tif]
